# The impact of early adversity on later life health, lifestyle, and cognition

**DOI:** 10.1186/s12889-024-20768-3

**Published:** 2024-11-27

**Authors:** Morgane Künzi, D. A. Gheorghe, J. Gallacher, S. Bauermeister

**Affiliations:** 1 Dementias Platform UK, Department of Psychiatry, University of Oxford, Warneford Hospital, Oxford, OX3 7JX UK; 2 Department of Experimental and Theoretical Neuroscience, Transylvanian Institute of Neuroscience, Cluj-Napoca, Romania

**Keywords:** Adversity, Physical health, Mental health, Lifestyle, Cognition

## Abstract

**Background:**

Early life adversity has been shown to have long-lasting detrimental effects on a variety of biopsychosocial outcomes. Early adversity and its consequences may directly or indirectly affect cognitive aging and increase the risk of developing dementia in older age. Investigating the biopsychosocial outcomes associated with early adverse experiences is essential to inform health policies and promote healthy cognitive development across the life course.

**Methods:**

The aim of this study is to investigate the effect of early adversity (i.e., abuse and deprivation) on selected outcomes (i.e., physical and mental health, lifestyle, and cognition) in two UK cohorts (the English Longitudinal Study of Ageing (ELSA), *N* = 12,653, *Mdn*_age_ = 66, *SD*_age_ = 9.58; UK Biobank, *N* = 502,360, *Mdn*_age_ = 58, *SD*_age_ = 8.09). In both cohorts, adversities were self-reported retrospectively, and only those adversity types assessed in both cohorts were utilized. A post-hoc analysis was performed to examine the role of education as a mediator of the association between early adversity and the selected outcomes.

**Results:**

Most of the results show that early adversity is negatively associated with health (both physical and mental), lifestyle, and cognition while also highlighting the important mediating role of education. However, differences exist according to the specific adversity experienced and the cohort studied.

**Conclusions:**

The results found bring into attention the complex associations between early adversity and multiple later life outcomes, and suggest that various mechanisms might be at play. Furthermore, the findings highlight the importance of multi-cohort comparisons for the generalization of the results.

**Supplementary Information:**

The online version contains supplementary material available at 10.1186/s12889-024-20768-3.

## Introduction

Early life adversity has been shown to have long-lasting detrimental effects on a variety of biopsychosocial outcomes (i.e., physical health, mental health, lifestyles, cognition, and brain atrophy) [1–16]. Multiple lines of evidence also show that early life adversity may directly or indirectly affect cognition and thus increase the risk of dementia (e.g., [17–23]). One proposed pathway suggests that early adversity may lead to the adoption of an unhealthy lifestyle as a way to cope with the adversity (or with the adversity consequences, e.g., depression), including overeating to compensate for stress-related hypothalamic-pituitary-adrenal (HPA) axis activation [4, 6, 16, 24–26]. These coping/compensatory strategies, may in turn, adversely affect cognition and increase the risk of dementia [17, 19–23].

However, far from being straightforward, some of the findings on the effects of early adversity suggest that not all adverse experiences have a negative effect on later life outcomes (i.e., cognition) [e.g., 27, 28]. Moreover, select results show that the effects and strength thereof are different according to the adversity experienced [11, 29] but also within the same adversity category (e.g., trauma) [e.g., 2, 4, 8, 30]. This reflects not only the lack of mechanistic understanding of the adversity effects, but also varying practices in the operationalization of the adversity concept which is broadly defined as: “highly stressful, and potentially traumatic, events or situations” [31]. In a broad definition, early adversity includes different events occurring at different developmental periods and encompassing different aspects, such as the severity, duration, chronicity, and co-occurrence (i.e., adversity characteristics) [11, 29, 31–35]. This mix of different types of adversities including different adversity characteristics may bias the interpretation of the adversity effects, leading to inconsistent results and increasing the difficulty of addressing the question regarding the underlying mechanisms. Indeed, although the disruption of the stress-response systems and allostatic load (stress-related wear and tear on the body), have been suggested as probable mechanisms, other pathways cannot be ruled out [36]. This might also explain some of the inconsistent findings in the literature. Multi-cohort comparisons and replications would assist in disentangling the divergent outcomes and could lead to a better understanding of the effects of adversity on biopsychosocial outcomes and the underlying mechanisms of these associations [37].

The main aim of this study was to investigate the effects of distinct, specific adversities within the same adversity category (i.e., abuse or deprivation) on selected (multiple) outcomes (i.e., physical health, mental health, lifestyles, and cognition) in two UK cohorts. The objective was to determine the effect of each specific adversity on the different outcomes studied, while considering multiple adversities simultaneously in a single model, to clarify the literature findings, and to better understand the negative effects of adversity. Disentangling and understanding these effects is the first necessary step toward a mechanistic understanding, which may support the development of effective hypothesis testing and ultimately, novel preventive and therapeutic interventions.

## Methods

### ELSA participants

This study included data from the English Longitudinal Study of Ageing (ELSA) [38]. ELSA is a longitudinal study that started in 2002 with follow-ups every 2 years to investigate changes over time in socioeconomic conditions, health, and cognitive functioning in participants aged 50 and older. In this study, early adversity data from wave 3 (2006–2007), physical and mental health data from waves 6 (2012–2013) and 7 (2014–2015), lifestyle data from wave 7, and cognitive data from wave 7 (2014–2015) were used (*N* = 12,653, *Mdn*_*age*_ = 66, *SD*_*age*_ = 9.58, 55.53% of the respondents were women). ELSA received ethical approval from the London Multi-Centre Research Ethics Committee on 27th October 2005 (05/MRE02/63) for wave 3 and from the NRES Committee South Central - Berkshire on 28th November 2012 (11/SC/0374) for wave 6 and 2013 (13/SC/0532) for wave 7. ELSA was conducted in accordance with the Declaration of Helsinki and all participants gave informed consent for their participation.

## UK Biobank participants

UK Biobank is a large population-based prospective cohort study that started in 2006 with numerous follow-ups continuing and additional data collected at each new wave. The participants were recruited across the United Kingdom, and sociodemographic, lifestyle, medical history, environmental, cognitive, and biomedical data were collected [39]. For this study, the data of 502,360 participants aged between 40 and 73 years (*Mdn*_*age*_ = 58, *SD*_*age*_ = 8.09, 54.40% of the participants were women) were used. Adversity data were collected with an online questionnaire. Sociodemographic and lifestyle data were collected through touchscreen questionnaires. Cognitive tasks were administered through an unsupervised computerized touchscreen interface. UK Biobank study received ethical approval from the Research Ethics Committee (approval letter dated 17th June 2011, Ref 11/NW/0382) and was conducted in accordance with the Declaration of Helsinki. All participants gave written informed consent for their participation.

### ELSA materials

#### Early adversity

Early adversity data were collected in wave 3 using a self-completion questionnaire. Participants were asked to indicate whether they experienced any of the following events and the age at which they first experienced the adversity: Victim of serious physical attack or assault (physical assault); Experienced sexual assault (rape or harassment; sexual assault); Physically abused by parents before the age of 16 (parental abuse). Variables physical assault and sexual assault were dichotomized and recoded as 0 = no experience of this adversity before the age of 16, 1 = experience of this adversity before the age of 16.

For childhood socioeconomic deprivation, participants had to answer three items relating to socioeconomic deprivation at the age of 10: (1) the number of bedrooms in the lived-in residence, (2) the number of people living in the residence, and (3) the number of books in the home. A principal-component factor analysis was performed to determine the items loading on the deprivation dimension to compute a deprivation variable. Based on the results of this analysis, only the items “number of bedrooms in the lived-in residence”, and “number of books in the home” were kept for the computation of a factor score. The factor score was then recoded as 0 (score between 2.5 and 100) and 1 (score between 0 and 2) to obtain a dichotomic deprivation variable (see Table 1 for the frequency table of the early adversity items).

**Table 1:** Frequency table of the early adversity items in the ELSA dataset

Early adversity	No	Yes	Missing
Physical assault	258 (75.88%)	82 (24.12%)	28,910
Sexual assault	6308 (96.44%)	233 (3.56%)	22,709
Parental abuse	6290 (96.52%)	227 (3.48%)	22,733
Deprivation	5078 (68.45%)	2341 (31.55%)	21,831

#### Physical and mental health

##### Body mass index (BMI)

The BMI was derived from height and weight measured in wave 6 during the nurse visit using the standard formula: weight (kg)/height (m2).

##### Self-reported health

Self-reported health was assessed in wave 7 with the following item: “Would you say your health is…” to answer on a 5-point Likert scale coded from 0 = poor to 4 = excellent.

##### Depression

Depressive symptoms were measured with the eight items version of the Centre for Epidemiologic Studies Depression Scale (CES-D) [40] in wave 7. A sum score was computed based on the number of CES-D questions answered “Yes”.

#### Lifestyles

##### Smoking status

The smoking status was collected with the item “Do you smoke cigarettes at all nowadays?” (coded 0 = No, 1 = Yes) asked in wave 7.

##### Alcohol

The alcohol frequency consumption was asked in wave 7 with the item “how often the respondent has an alcoholic drink during the last 12 months” coded from 0 = not at all in the last 12 months to 7 = almost every day.

#### Cognition

Immediate memory was assessed in wave 7 with a list of 10 words to immediately recall.

Executive functions were assessed in wave 7 with a verbal fluency task consisting in naming as many different animals as possible in 60 s.

#### Control variables

Age and education were used as control variables. The age variable from wave 7 was used. For the educational qualification obtained, information from the previous and current waves collected was merged and used as an education measure coded as 1 = No qualification, 2 = NVQ1, CSE or other grade equivalents, 3 = NVQ2 or O-level GCE equivalent, 4 = NVQ3 or A-level GCE equivalent, 5 = higher education below degree, and 6 = NVQ4, NVQ5 or equivalent (see Table 2 for the descriptive statistics of the outcomes and control variables).

**Table 2:** Descriptive statistics of the outcomes and control variables in the ELSA dataset

Variables	*n*	*Mdn*	*SD*	*Min*	*Max*
BMI	7651	27.54	5.11	15.10	54.60
Self-rated health	8897	2	1.10	0	4
Depression	9069	1	1.85	0	8
Smoking	6079	0	0.38	0	1
Alcohol	7918	4	2.18	0	7
Immediate memory	8877	6	1.84	0	10
Verbal fluency	8900	21	7.32	0	67
Age	9440	66	9.58	43	89
Education	8160	3	1.87	1	6

*Note. n* = Number of respondents, *Mdn* = Median, *SD* = Standard Deviation, *Min* = Minimum range value, and *Max* = Maximum range value

### UK Biobank materials

#### Early adversity

Early adversity items were based on the Childhood Trauma Questionnaire (CTS-5) [41]: i.e., I felt that someone in my family hated me (emotional abuse); Someone molested me sexually (sexual abuse); I felt loved as a child (emotional neglect); People in my family hit me so hard that it left me with bruises or marks (physical abuse); There was someone to take me to the doctor if I needed it (physical neglect). The data was collected between 2016 and 2017 through the mental health online questionnaire. Participants had to answer these questions on a 5-point Likert scale: never true, rarely true, sometimes true, often true, very often true. Physical neglect and emotional neglect were reversed coded, and all the variables were dichotomized and recorded as 0 = never having experienced the adverse event, and 1 = having experienced the adverse event (for a similar procedure see Gheorghe et al., 2021 [7]; see Table 3 for the frequency table of the early adversity items).

**Table 3:** Frequency table of the early adversity items in the UK Biobank dataset

Early adversity	No	Yes	Missing
Physical neglect	130,510 (83.54%)	25,713 (16.46%)	346,140
Sexual abuse	141,810 (91.23%)	13,636 (8.77%)	346,917
Emotional neglect	81,556 (52.06%)	75,113 (47.94%)	345,694
Physical abuse	127,162 (81.03%)	29,777 (18.97%)	345,424
Emotional abuse	132,316 (84.36%)	24,528 (15.64%)	345,519

#### Physical and mental health

##### BMI

BMI was derived from height and weight measured in wave 3 (2019–2021) using the standard formula: weight (kg)/height (m^2^).

##### Self-reported health

Self-reported health was assessed with the following item: “In general how would you rate your overall health” to answer on a 4-point scale coded from 0 = poor to 3 = excellent. This variable was assessed four times, but only the variable from the most recent assessment (wave 3; 2019–2021) was selected for the analysis.

##### Depression

Depressive symptoms were measured between 2016 and 2017 with the Patient Health Questionnaire-9 questions (PHQ-9) [42, 43]. Participants had to answer how often they have been bothered by a list of problems on a scale from 0 not at all to 3 nearly every day. Based on the answers to these 9 items, a sum score was computed to obtain a depression variable score. A LN + 1 transformation was applied given the right skewness in the data.

#### Lifestyles

##### Smoking status

The smoking status was collected with the item “Do you smoke tobacco now?” recoded as 0 = No or 1 = Yes. Only the item from wave 3 (2019–2021) was selected for the analysis.

##### Alcohol

The alcohol intake frequency was collected with the following item “About how often do you drink alcohol?” coded from 0 = Never to 5 = Daily or almost daily. Only the item from wave 3 (2019–2021) was selected for the analysis.

#### Cognition

Visual declarative memory was measured between 2014 and 2015 with a ‘pairs-matching’ test. Participants were shown 3, 6, and 8 pairs of cards and had to memorize the cards and their positions to match them by memory. The variable used corresponds to the number of errors each participant made. Thus, a higher number represents a lower performance. Following the procedure of Lyall et al., 2016 [44] the round corresponding to 6 pairs was selected (due to the greater scope of score variation) and given the right skewness in the data, a LN + 1 transformation was applied.

Executive function was assessed with the Trail Making Test (TMT) Part A (TMTA) and B (TMTB) between 2014 and 2015. TMTA is the time in seconds to correctly connect the numbers 1 to 25 in ascending order, whereas part B (TMTB) represents the time in seconds to correctly connect the numbers 1 to 13 and the letters A to L in ascending and alphabetic order alternating between number and letter (1-A, 2-B, 3-C,.,12-L-13). Only the TMTB was selected as it is a measure of executive functioning, through the switching component [45]. As the TMTB variable was not normally distributed a log transformation was applied (see Fawn-Ritchie & Deary, 2020 [45] for a similar procedure).

Verbal and numerical reasoning was assessed using the Fluid Intelligence Test between 2014 and 2015. In this task, participants were given 2 min to correctly solve as many logic and reasoning problems as possible. The number of correct problems solved is the variable used.

Processing speed was measured with a reaction time (RT) task named “snap game” (https://biobank.ctsu.ox.ac.uk/crystal/crystal/docs/Snap.pdf) including 12 rounds (0–11). In this task, participants were shown two cards and had to press the button to indicate a match between the two symbols of the displayed cards. The RT is the time interval (ms) between the display of the two cards and the button pressed (no matter the correctness of the match). Only the RT assessment from wave 3 (2019–2021) was selected for the analysis. Training rounds (0–4), RT under 50ms, and RT over 200ms were excluded from the analysis [46, 47]. Note that no additional participants were required to be excluded since none had an RT greater than 3 standard deviations. Following the procedure in Künzi et al. (2022) [47] an RT mean was computed for participants having at least four rounds without missing data. The RT mean was log-transformed due to the non-normal distribution of the data (see Lyall et al., 2016 [44] for a similar procedure).

#### Control variables

Age at the recruitment and the number of years of education were used as control variables. Missing data on the education variable were imputed with the variable “qualifications achieved” (see Table 4 for the descriptive statistics of the outcomes and control variables).

**Table 4:** Descriptive statistics of the outcomes and control variables in the UK Biobank dataset

Variables	*n*	*Mdn*	*SD*	*Min*	*Max*
BMI	5355	25.83	4.38	13.88	51.61
Self-rated health	5353	2	0.66	0	3
Depression without transformation	154,302	2	3.69	0	27
Depression with transformation	154,302	1.10	0.83	0	3.33
Smoking	5357	0	0.15	0	1
Alcohol	5360	3	1.40	0	5
Visual episodic memory without transformation	118,496	4	3.12	0	45
Visual episodic memory with transformation	118,496	1.61	0.62	0	3.83
TMTB without transformation	103,999	61.18	25.75	20.56	746.53
TMTB with transformation	103,999	4.11	0.34	3.02	6.62
Fluid intelligence	123,579	6	2.06	0	14
Processing speed without transformation	5043	573.5	107.11	306.83	1755
Processing speed with transformation	5043	6.35	0.17	5.73	7.47
Age	502,360	58	8.09	40	73
Education	495,645	17	2.77	5	35

*Note. n* = Number of respondents, *Mdn* = Median, *SD* = Standard Deviation, *Min* = Minimum range value, and *Max* = Maximum range value

### ELSA statistical analysis

**Fig. 1:**
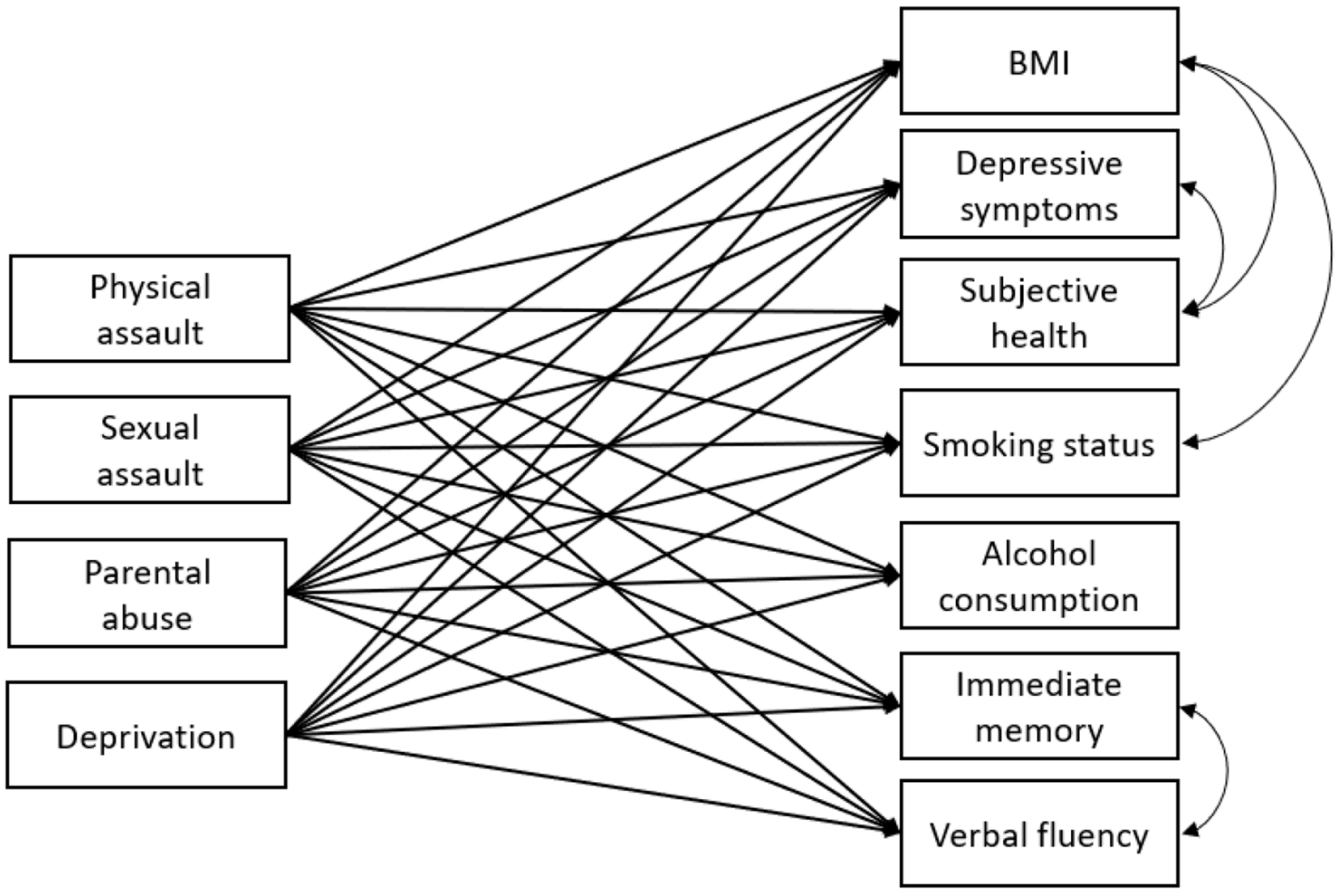
Simplified Illustration of the Model Fitted in the ELSA Dataset. *Note.* For clarity purposes, the control variables and covariances between adversity items were not drawn

Path analysis was performed in STATA V.17.0 (StataCorp, College Station, TX, USA) with α = 0.01 to limit type I error given the large sample size [48]. The model was defined as the following: Physical abuse, sexual abuse, parental abuse, and deprivation predicted BMI, self-reported health, depression, smoking, alcohol, memory, and verbal fluency. The error terms of BMI and self-rated health, the error terms of BMI and smoking, the error terms of depressive symptoms and self-rated health, as well as the error terms of memory and verbal fluency were correlated [44, 45, 49–52]. For the control variables, age predicted BMI, self-reported health, depression, memory, and verbal fluency observed variables. Education predicted the same variables as age but with smoking status and alcohol consumption added (see Fig. 1 for a simplified illustration of the model tested). The Full Information Maximum Likelihood (FIML) estimation method was used. The model’s goodness of fit was assessed with the comparative Fit Index (CFI; >= 0.95) and the Root Mean Squared Error of Approximation (RMSEA; < 0.06) [53, 54]. Supplementary analyses were also performed to examine the associations between adversity variables (see supplements Table A to Table F), sex differences (see supplements Table G and Table H), and the role of education as a mediator of the association between early adversity and the selected outcomes (see supplements Table I and Table J).

### UK Biobank statistical analyses

**Fig. 2:**
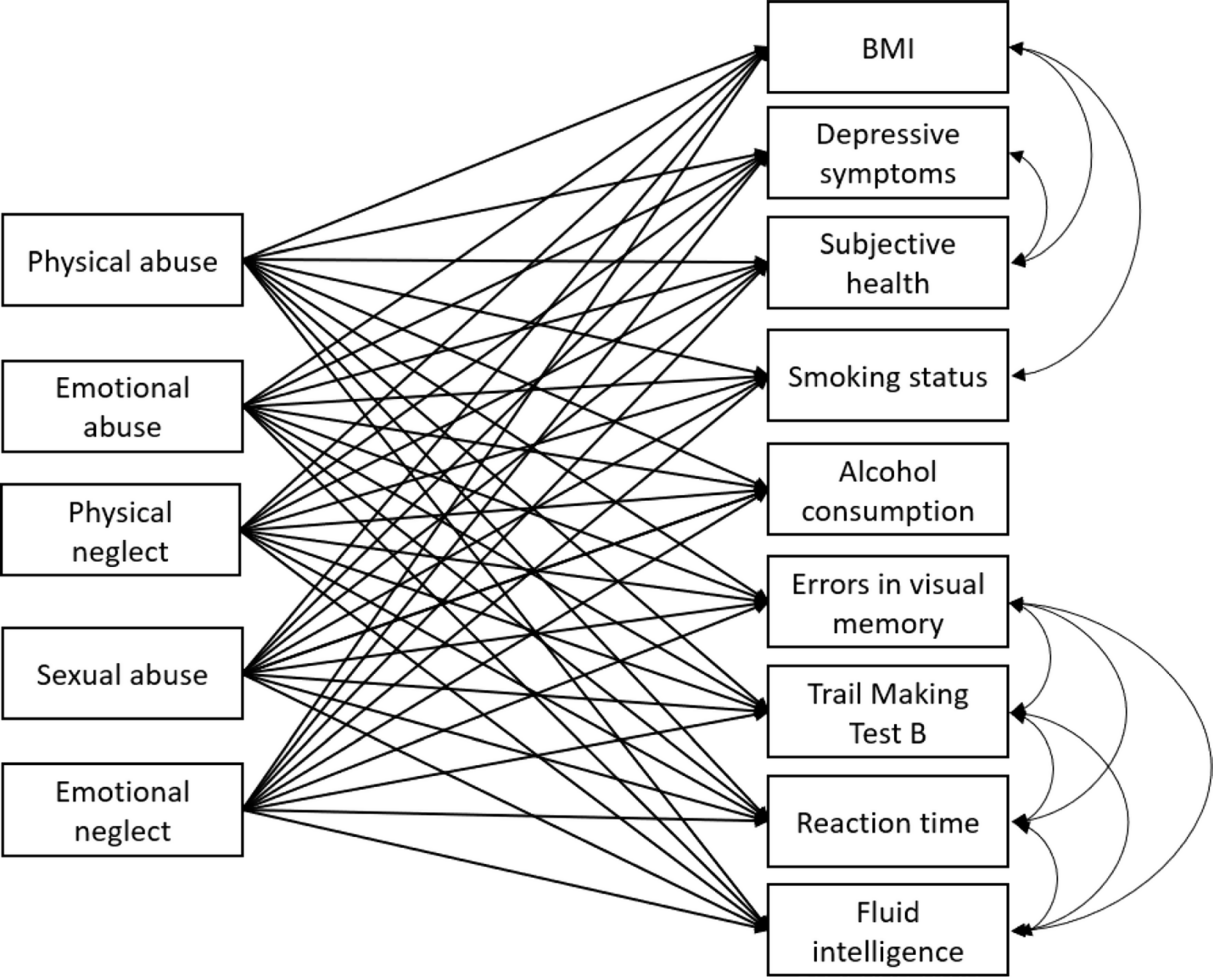
Simplified Illustration of the Model Fitted in the UK Biobank Dataset. *Note.* For clarity purposes, the control variables were not drawn

Participants aged lower than 40 were excluded from our analysis (*n* = 7), and similarly, a path analysis was performed considering α = 0.01. The model was defined as the following: Physical neglect, sexual abuse, emotional neglect, physical abuse, emotional abuse predicted BMI, self-reported health, depression, smoking, alcohol, visual memory, executive functioning (i.e., switching), processing speed, and fluid intelligence. The error terms of BMI and self-rated health, the error terms of BMI and smoking, the error terms of depressive symptoms and self-rated health, as well as the error terms of each cognitive task, were correlated [44, 45, 49–52]. For the control variables, age predicted BMI, self-reported health, depression, visual memory, executive functioning (i.e., switching), processing speed, and fluid intelligence observed variables. Education predicted the same variables as age but with smoking status and alcohol consumption added (see Fig. 2 for a simplified illustration of the model tested). In line with the analysis in ELSA, the FIML estimation method was used and goodness of fit was assessed. Supplementary analyses were also performed to examine the associations between adversity variables (see supplements Table K to Table T), sex differences (see supplements Table U and Table V), and the role of education as a mediator of the association between early adversity and the selected outcomes (see supplements Table W and Table X).

## Results

### ELSA dataset

There was no multicollinearity between the exogenous variables of the model (i.e., physical assault, sexual assault, parental abuse, deprivation, age, and education) as indicated by the variance inflation factor (VIF) values equal or lower than 1.15. The model fit was very good CFI = 0.956, and RMSEA = 0.041 [53, 54]. Table 5 shows the standardized coefficients of the model.

**Table 5:** Standardized coefficients of the Model in the ELSA dataset

ELSA Dataset						
Outcomes	Early adversity				Control variables	
	Physical assault	Sexual assault	Parental abuse	Deprivation	Age	Education
BMI	− 0.178**	0.042*	0.052*	− 0.013	− 0.037	− 0.145**
Subjective health	0.436**	− 0.067**	− 0.119**	− 0.020	− 0.243**	0.215**
Depressive symptoms	− 0.349**	0.079**	0.112**	0.045	0.093**	− 0.145**
Smoking status	− 0.335**	0.056*	0.066**	0.021	-	− 0.114**
Alcohol consumption	0.291**	− 0.071**	− 0.074**	− 0.031	-	0.255**
Immediate memory	0.245**	− 0.008	− 0.051**	− 0.056*	− 0.335**	0.237**
Verbal fluency	0.247**	− 0.012	− 0.042*	− 0.082**	− 0.283**	0.232**

** *p*-value < or = 0.001, * *p*-value < or = 0.01

#### Physical assault

Physical assault before the age of 16 significantly predicted lower BMI, higher self-rated health, lower levels of depressive symptoms, being non-smoker, higher frequency of alcohol consumption, better immediate memory and verbal fluency performance.

#### Sexual assault

Sexual assault before the age of 16 significantly predicted higher BMI, lower self-rated health, higher levels of depressive symptoms, being smoker, lower frequency of alcohol consumption. No significant associations were found between sexual assault and immediate memory as well as verbal fluency performance.

#### Parental abuse

Physical abuse from parents before the age of 16 significantly predicted higher BMI, lower self-rated health, higher levels of depressive symptoms, being smoker, lower frequency of alcohol consumption, and poorer immediate memory and verbal fluency performance.

#### Deprivation

Deprivation at age 10 significantly predicted poorer immediate memory and verbal fluency performance. No significant associations were found between deprivation and the other outcomes of interest (i.e., BMI, subjective health, depressive symptoms, smoking status, and alcohol consumption).

#### Control variables

Being older significantly predicted lower self-rated health, higher levels of depressive symptoms, and poorer immediate memory and verbal fluency performance. No significant association was found between age and BMI. Having achieved higher educational qualifications significantly predicted lower BMI, higher self-rated health, lower levels of depressive symptoms, being non-smoker, higher frequency of alcohol consumption, and better immediate memory and verbal fluency performance.

### UK Biobank dataset

There was no multicollinearity between the exogenous variables of the model (i.e., Physical neglect, sexual abuse, emotional neglect, physical abuse, emotional abuse, age, and education) as indicated by the VIF values equal or lower than 1.22. The model fit was very good CFI = 0.996, and RMSEA = 0.004 [53, 54]. Table 6 shows the standardized coefficients of the model.

**Table 6:** Standardized coefficients of the Model in the UK Biobank dataset

UKB Dataset							
Outcomes	Early adversity					Control variables	
	Physical abuse	Emotional abuse	Physical neglect	Sexual abuse	Emotional neglect	Age	Education
BMI	0.041	0.023	0.019	− 0.003	0.018	− 0.056**	− 0.130**
Subjective health	0.005	− 0.066**	− 0.013	− 0.027	− 0.077**	0.028	0.088**
Depressive symptoms	0.033**	0.133**	0.013**	0.067**	0.078**	− 0.145**	− 0.048**
Smoking status	0.002	0.040	− 0.001	− 0.024	0.004	-	0.006
Alcohol consumption	− 0.015	− 0.030	− 0.052*	− 0.025	0.007	-	0.062**
Errors in visual memory	− 0.004	0.002	0.021**	0.006	0.001	0.128**	− 0.015**
Trail Making Test (Switching)	0.0004	0.004	0.096**	0.012**	0.004	0.369**	− 0.163**
Reaction time	− 0.023	0.034	0.026	0.037	− 0.015	0.346**	− 0.076**
Fluid intelligence	− 0.004	0.008	− 0.112**	− 0.011**	0.006	− 0.071**	0.307**

** *p*-value < or = 0.001, * *p*-value < or = 0.01

#### Physical abuse

Physical abuse in childhood significantly predicted higher levels of depressive symptoms later in life. No significant associations were found between physical abuse and the other outcomes of interest (i.e., BMI, subjective health, smoking status, alcohol consumption, errors in visual memory, TMTB completion time, RT, and performance in fluid intelligence).

#### Emotional abuse

Emotional abuse in childhood significantly predicted lower self-rated health and higher levels of depressive symptoms later in life. No significant associations were found between emotional abuse and the other outcomes of interest (i.e., BMI, smoking status, alcohol consumption, errors in visual memory, TMTB completion time, RT, and performance in fluid intelligence).

#### Physical neglect

Physical neglect in childhood significantly predicted higher levels of depressive symptoms, lower frequency of alcohol consumption, more errors in the visual memory task, slower TMTB completion time, and lower performance in fluid intelligence later in life. No significant associations were found between physical neglect and the other outcomes of interest (i.e., BMI, subjective health, smoking status, and RT).

#### Sexual abuse

Sexual abuse in childhood significantly predicted higher levels of depressive symptoms, slower TMTB completion time, and poorer performance in fluid intelligence later in life. No significant associations were found between sexual abuse and the other outcomes of interest (i.e., BMI, subjective health, smoking status, alcohol consumption, errors in visual memory, and RT).

#### Emotional neglect

Emotional neglect in childhood significantly predicted lower self-rated health and higher levels of depressive symptoms later in life. No significant associations were found between emotional neglect and the other outcomes of interest (i.e., BMI, smoking status, alcohol consumption, errors in visual memory, TMTB completion time, RT, and performance in fluid intelligence).

#### Control variables

Being older significantly predicted a lower BMI, lower levels of depressive symptoms, more errors in the visual memory task, more time to complete the TMTB, slower RT, and poorer performance in fluid intelligence later in life. No significant association was found between age and subjective health. More education significantly predicted lower BMI, higher self-rated health, lower levels of depressive symptoms, higher frequency of alcohol consumption, fewer errors in the visual memory task, faster TMTB completion time, faster RT, and better performance in fluid intelligence later in life. No significant association was found between years of education and smoking status.

## Discussion

### ELSA dataset

In the ELSA dataset, most of the early adversities (with the exception of sexual assault, which was not associated with cognition, and deprivation, which was only associated with cognition) were associated with BMI, subjective health, depressive symptoms, smoking status, alcohol consumption, and cognition (i.e., immediate memory and verbal fluency). Specifically, in line with the literature, early adversities were found to be adversely associated with BMI, subjective health, depressive symptoms, smoking status, alcohol consumption, and cognition [2–6, 9–16, 55]. However, some inconsistencies were also found.

First, contrary to its counterpart adversities (i.e., physical assault and parental abuse) having experienced sexual assault before the age of 16 was not associated with immediate memory and verbal fluency performances. These results may either suggest a robustness of immediate memory and verbal fluency to the negative impact of experiencing early sexual abuse or a fully mediated effect of experiencing early sexual abuse on cognition via mental health, lifestyle, and/or education. Having experienced physical assault before the age of 16 was associated with lower BMI, lower levels of depressive symptoms, being non-smoker, higher self-rated health, and higher cognitive performance, but physical assault was also associated with higher frequency of alcohol consumption. These results need special focus and further study to be able to fully grasp them. However, different explanations could be suggested. The low response rate to this specific adversity (*n* = 340) may bias the results found (i.e., statistical power) but may also suggest that the way this adversity was assessed was not clearly formulated or added confusion in relation to the other adversities assessed and their possible overlap (see supplements Table A, Table B, and Table D). Another explanation could be the role of chronicity. No information regarding chronicity was available in this dataset, therefore it could not be excluded that the respondents had, in majority, experienced a single episode of physical assault which may therefore contribute to the development of their resilience - achieving positive outcomes despite the experience of adversity [56]– [57].

A positive association was found between physical assault and increased alcohol consumption, contrary to the experience of sexual assault and parental abuse which were both associated with lower alcohol consumption. Interestingly, in the literature low-to-moderate alcohol consumption has been positively associated with cognition and protective against cognitive decline [58–60]. However, some caution is needed when interpreting the results related to alcohol consumption as chronic and heavy alcohol consumption has a negative impact on health and cognition and any positive effect of low-to-moderate alcohol consumption may also be outweighed by the risk of harmful effects [58–60].

Regarding the experience of deprivation, only two associations with cognition were found to be significant, poorer immediate memory and verbal fluency performance. This result is in line with the literature showing a negative effect of low childhood socioeconomic status with later life cognition [61–65]. The finding that deprivation has no significant effect on physical and mental health, neither lifestyle variables, may be due to the severity of the other adversities included in the model and the often-found co-occurrence between adversities (of different severities) which could thus outweigh the potential negative effect of deprivation [8, 11]. Another explanation could be that deprivation only indirectly affects physical and mental health, as well as lifestyle, and thus that other factors are at play in these associations (e.g., education).

### UK Biobank dataset

In the UK Biobank dataset, experiencing physical abuse, emotional abuse, physical neglect, sexual abuse, or emotional neglect was associated with depressive symptoms as previously demonstrated in the literature [2, 3, 5, 11–13, 15]. In line with studies showing a negative effect of early adversity on subjective health [66–68], experiencing emotional abuse or emotional neglect showed a similar pattern of results as both were associated with higher levels of depressive symptoms and lower self-rated health. This similar pattern of results may suggest the involvement of a common mechanism related to the emotional component of adversity in contrast to the literature suggesting a common mechanism related to the threat or deprivation component of adversity [36, 55, 69]. Interestingly, and contrary to the literature findings, all other associations between emotional abuse or emotional neglect and the other outcomes of interest were non-significant [4–6, 10, 11, 14, 16, 55]. It may be possible that the experience of emotional adversity may have a strong influence on self-perception/self-esteem but also on personal control (e.g., subjective health and self-reported depressive symptoms) that may indirectly affect the objective measures (e.g., BMI, and cognition), contrary to physical adversity (neglect or sexual abuse) which shows a direct effect on objective cognitive measures [66, 70, 71]. Nonetheless, further studies including mediation links through self-perception/self-esteem as well as the addition of self-perceived cognitive measures may provide a better insight into these results.

For physical adversity (physical abuse, physical neglect, and sexual abuse) no specific pattern of outcomes linked to the physical component could be identified (all the early adversities were associated with depressive symptoms). Interestingly, although, both physical neglect and sexual abuse showed an association with higher levels of depression and slower TMTB completion time, physical neglect showed the most significant associations with the outcomes studied. Physical neglect was associated with higher levels of depressive symptoms, lower frequency of alcohol consumption, and poorer cognitive performance (i.e., visual memory task, TMTB, and fluid intelligence). These results are in line with the literature showing a negative effect of early adversity on mental health, and cognition (e.g., [2, 3, 5, 10–13, 15]). However, the findings do not support previous research showing a negative effect of early adversity on physical health and lifestyle (e.g., [3, 9, 11, 12, 16]). The role of a mediator may be hypothesized as underlying the association between physical neglect and physical health, as well as smoking status. Interestingly, the results regarding alcohol consumption need to be cautiously interpreted due to some positive associations found in the literature between alcohol consumption and health and cognition [58–60].

Early sexual abuse was associated with cognitive tasks involving executive functions (i.e., trail-making test and fluid intelligence), suggesting that early sexual abuse may have an effect on the prefrontal and frontal cortices. Importantly the effect of sexual abuse on grey matter volume in the frontal cortex has been demonstrated in a specific time period (i.e., 14–16 years old) [72]. Therefore, it may be possible that sexual abuse, in this sample, may have been experienced specifically in this time frame, which may also explain the absence of a significant association between sexual abuse and memory (i.e., hippocampus) in this sample.

Physical abuse was only significantly associated with higher levels of depressive symptoms. In general, the non-significant associations between adversity and the outcomes of interest may be explained by education (except for smoking status). Indeed, education was significantly associated with all the outcomes of interest except smoking status. Therefore, it may be possible that education in part or fully mediates the association between adversity and the outcome of interest. Interestingly, all early adversities were not significantly associated with BMI and smoking status contrary to the literature findings and the ELSA dataset. Although it is possible that the effect of early adversity on BMI is mediated by education (in contrast to smoking status), for smoking status, it is important to highlight that in the UK biobank dataset, a high number of missing values were present in this variable (i.e., missing values = 497,006), and in addition, only a few participants smoked at the assessment time (*n* = 118). This may, in part, explain why no significant association was found with the smoking variable but also the differences found between these two cohorts for smoking. In addition, all early adversities were also not significantly associated with reaction time. This result may be explained by the significant association found between age and reaction time. Indeed, age is known to affect reaction time, with increasing age slower reaction time, and thus age may explain most of the reaction time variance in this model [73, 74].

### Cohort comparisons

Overall, and in both cohorts, most of the early adversities studied were found to be negatively associated with physical health, mental health, lifestyle, and cognition. Importantly, in both cohorts, differences according to the specific adversity experienced were found, in line with the literature suggesting the importance of considering the specific adversity experienced and the potential role of in part differential underlying mechanisms [32, 36]. In addition, across the two cohorts, almost all early adversities (i.e., physical assault, sexual assault, and parental abuse in the ELSA dataset and physical abuse, emotional abuse, physical neglect, sexual abuse, and emotional neglect in the UK Biobank dataset) were shown to be consistently associated with depressive symptoms. In line with the literature, except for physical assault in the ELSA dataset, each adversity experience was associated with higher levels of depressive symptoms [2, 3, 5, 11–13, 15]. The same arguments as the ones previously mentioned may explain this result (i.e., low response rate, the role of chronicity, and increased resilience). Importantly, physical assault in the ELSA dataset has no adversity equivalent in the UK Biobank, since physical abuse is closer to parental abuse. Although the early adversities have been cautiously selected in both cohorts to be the most comparable, there is no perfect overlap between them with the greatest overlap between parental abuse (ELSA) and physical abuse (UK Biobank) as well as sexual assault (ELSA) and sexual abuse (UK Biobank).

The other discrepancies observed between the two cohorts may be determined by the different assessments (i.e., adversity and outcomes assessments). Cohort characteristics (i.e., education but also age) may also explain these results mainly for subjective health and cognition. Indeed, the ELSA cohort is older, and therefore decrease in subjective health and cognitive impairment may be more detectable in an older population.

### Mediation

Education was associated with numerous outcomes in both cohorts, therefore education may (fully and partially) mediate the effects of early adversity on the outcomes of interest in both cohorts [9]. Importantly, education has been found to be important for resilience [75]. To test this hypothesis, additional analyses were performed with education as a mediator. The supplementary analyses support this mediation explanation. In the ELSA dataset, all the associations between deprivation and the outcomes of interest (i.e., BMI, subjective health, depressive symptoms, smoking status, alcohol consumption, immediate memory, and verbal fluency) are mediated by education. The associations between early adversities (i.e., physical assault, sexual assault, and parental abuse) and the outcomes of interest are not significantly mediated by education (see supplements Table I and Table J). In the UK Biobank, all the early adversities (i.e., physical abuse, emotional abuse, physical neglect, sexual abuse, and emotional neglect) are mediated by education for all of the outcomes of interest (i.e., BMI, subjective health, depressive symptoms, alcohol consumption, visual memory, Trail Making Test B, reaction time, and fluid intelligence) except for smoking status (see supplements Table W and Table X). These results, emphasize the mediating role of education in the associations between adversities (in general including deprivation and abuse) and physical health, mental health, lifestyle, and cognition.

### Strength and limits

This study has the advantage of using large sample sizes, which may be more representative of the general population, as well as cross-cohort models, which may increase the generalization of the effects found [76]. Therefore, at minimum, the associations between early adversities and depressive symptoms (measured differently according to the cohorts) seem robust.

However, in this study, the early adversities selected, although close, are not perfectly overlapping. Therefore, further studies with a perfect overlap between the cohort adversities would not only strengthen the association found with depression, but also replicate and thus strengthen the other results found in this study. It is also important that future studies use different cohorts from different countries and with different characteristics to generalize the results found. In addition, the inclusion of longitudinal (instead of cross-sectional) data would provide access to temporality and insight into the causal relationship between the variables of interest. Furthermore, the lack of consistency in the adversity assessment across the studies makes the replicability and, thus, generalization of the results difficult. In addition, the population included in these cohorts and the adversity assessment (self-reported and retrospective) are subject to various biases (e.g., survival, selection, and resilience bias, recall, social and mental health bias) [77–83]. However, it is important to note that the reliability of self-reported adversity has already been emphasized and might be even under-reported [84, 85].

## Conclusion

This research aims to inform further studies into the associations between early adversity and physical health, mental health, lifestyle, and cognition. The results suggest that most of the adversities are negatively associated with physical health, mental health, lifestyle, and cognition, although some exceptions remain. Indeed, the results differ according to the specific early adversity experienced and the outcome studied, suggesting in part the involvement of different underlying mechanisms related to the specific adversity experienced and the outcome studied. Importantly, in the associations between early adversity and physical health, mental health, lifestyle, and cognition the mediating role of education has been highlighted. In addition, although the cohort comparison suggests that early adversity, in general, is associated with higher levels of depressive symptoms, it also highlighted cohort differences and the need for consistency across studies primarily in the adversity assessment but also in the outcomes investigated. This consistency is important for enabling future studies to uncover the underlying mechanisms, by revealing the key role of certain adversity components, potentially including the emotional component, as suggested by the results of the present study.

It is therefore essential to continue to study the associations between specific early adversities experienced and various outcomes among different cohorts in order to better understand these associations and to be able to uncover the underlying mechanisms behind these associations with the ultimate goal of promoting resilience through new interventions.

## Electronic supplementary material

Below is the link to the electronic supplementary material.

Supplementary Material 1

## Data Availability

The data used in this study came from the English Longitudinal Study of Ageing (ELSA; https://www.elsa-project.ac.uk/) and the UK Biobank (http://www.ukbiobank.ac.uk) application number 15697 (PI John Gallacher). Access to the ELSA data can be requested through the ELSA website (https://www.elsa-project.ac.uk/accessing-elsa-data). Access to the UK Biobank data can be requested through the UK Biobank website (https://www.ukbiobank.ac.uk/enable-your-research/apply-for-access).

## Abbreviations

HPA: Hypothalamic-Pituitary-Adrenal
ELSA: English Longitudinal Study of Ageing
BMI: Body Mass Index
CES-D: Epidemiologic Studies Depression Scale
CTS-5: Childhood Trauma Questionnaire
PHQ-9: Patient Health Questionnaire-9
TMT: Trail Making Test
TMTA: Trail Making Test Part A
TMTB: Trail Making Test Part B
RT: Reaction time
FIML: Full Information Maximum Likelihood
CFI: Comparative Fit Index
RMSEA: Root Mean Squared Error of Approximation
VIF: Variance Inflation Factor
