# Supplementary material for: The impact of early adversity on later life health, lifestyle, and cognition
Source: BMC Public Health. 2024 Nov 27;24:3294. doi: 10.1186/s12889-024-20768-3 (PMC11600748; doi:10.1186/s12889-024-20768-3)
Supplement: Supplementary file 1 — Supplementary Material 1 [file 12889_2024_20768_MOESM1_ESM.docx]

**Table A**

*Cross Tabulation and Chi^2^ Independence Test between Physical Assault and Parental Abuse in the ELSA Dataset*

| **Physical assault** | **Parental abuse** | |  |
| --- | --- | --- | --- |
|  | No | Yes | Total |
| No | 236 | 19 | 255 |
| Yes | 56 | 25 | 81 |
| Total | 292 | 44 | 336 |
| Pearson chi^2^(1) = 29.61, *p*-value < .001 | | | |

**Table B**

*Cross Tabulation and Chi^2^ Independence Test between Physical Assault and Sexual Assault in the ELSA Dataset*

| **Physical assault** | **Sexual assault** | |  |
| --- | --- | --- | --- |
|  | No | Yes | Total |
| No | 241 | 15 | 256 |
| Yes | 53 | 26 | 79 |
| Total | 294 | 41 | 335 |
| Pearson chi^2^(1) = 41.13, *p*-value < .001 | | | |

**Table C**

*Cross Tabulation and Chi^2^ Independence Test between Sexual Assault and Parental Abuse in the ELSA Dataset*

| **Sexual assault** | **Parental abuse** | |  |
| --- | --- | --- | --- |
|  | No | Yes | Total |
| No | 6,076 | 173 | 6,249 |
| Yes | 190 | 39 | 229 |
| Total | 6,266 | 212 | 6,478 |
| Pearson chi^2^(1) = 141.95, *p*-value < .001 | | | |

**Table D**

*Cross Tabulation and Chi^2^ Independence Test between Physical Assault and Deprivation in the ELSA Dataset*

| **Physical assault** | **Deprivation** | |  |
| --- | --- | --- | --- |
|  | No | Yes | Total |
| No | 175 | 76 | 251 |
| Yes | 56 | 20 | 76 |
| Total | 231 | 96 | 327 |
| Pearson chi^2^(1) = 0.44, *p*-value = .506 | | | |

**Table E**

*Cross Tabulation and Chi^2^ Independence Test between Sexual Assault and Deprivation in the ELSA Dataset*

| **Sexual assault** | **Deprivation** | |  |
| --- | --- | --- | --- |
|  | No | Yes | Total |
| No | 4,084 | 1,884 | 5,968 |
| Yes | 151 | 68 | 219 |
| Total | 4,235 | 1,952 | 6,187 |
| Pearson chi^2^(1) = 0.03, *p*-value = .871 | | | |

**Table F**

*Cross Tabulation and Chi^2^ Independence Test between Parental Abuse and Deprivation in the ELSA Dataset*

| **Parental abuse** | **Deprivation** | |  |
| --- | --- | --- | --- |
|  | No | Yes | Total |
| No | 4,091 | 1,874 | 5,965 |
| Yes | 139 | 68 | 207 |
| Total | 4,230 | 1,942 | 6,172 |
| Pearson chi^2^(1) = 0.19, *p*-value = .662 | | | |

**Table G**

*Standardized Coefficients of the Model for Male ELSA*

| **ELSA Dataset** |  | | | |  | |
| --- | --- | --- | --- | --- | --- | --- |
| **Outcomes** | **Early adversity** | | | | **Control variables** | |
|  | Physical assault | Sexual assault | Parental abuse | Deprivation | Age | Education |
| BMI | -.075 | -.005 | .047 | -.027 | -.080** | -.122** |
| Subjective health | .412** | -.040 | -.121** | .008 | -.249** | .256** |
| Depressive symptoms | -.362** | .035 | .112** | .034 | .108** | -.168** |
| Smoking status | -.347** | .038 | .126** | -.002 | - | -.115** |
| Alcohol consumption | .288** | -.029 | -.060 | -.051 | - | .197** |
| Immediate memory | .276** | -.046 | -.081** | -.064 | -.377** | .261** |
| Verbal fluency | .259** | -.061 | -.036 | -.087* | -.301** | .201** |
| ** *p*-value < or = .001, * *p*-value < or = .01  *N* = 4,297  RMSEA = .049  CFI = .950 | | | | | | |

**Table H**

*Standardized Coefficients of the Model for Female ELSA*

| **ELSA Dataset** |  | | | |  | |
| --- | --- | --- | --- | --- | --- | --- |
| **Outcomes** | **Early adversity** | | | | **Control variables** | |
|  | Physical assault | Sexual assault | Parental abuse | Deprivation | Age | Education |
| BMI | -.261** | .069* | .020 | .033 | -.043 | -.137** |
| Subjective health | .473** | -.074* | -.096** | -.060 | -.203** | .151** |
| Depressive symptoms | -.323** | .074* | .093** | .084* | .063* | -.075 |
| Smoking status | -.283** | .051 | .023 | .041 | - | -.085* |
| Alcohol consumption | .341** | -.068* | -.065* | -.069 | - | .221** |
| Immediate memory | .250** | -.003 | -.027 | -.028 | -.282** | .238** |
| Verbal fluency | .236** | .021 | -.035 | -.079* | -.244** | .241** |
| ** *p*-value < or = .001, * *p*-value < or = .01  *N* = 5,365  RMSEA = .046  CFI = .960 | | | | | | |

**Table I**

*Standardized Coefficients of the Mediation Model in the ELSA Dataset*

| **ELSA Dataset** |  | | | |  | |  |
| --- | --- | --- | --- | --- | --- | --- | --- |
| **Outcomes** | **Early adversity** | | | | **Control variables** | |  |
|  | | Physical assault | Sexual assault | Parental abuse | Deprivation | Age | Education |
| BMI | -.18** | .04* | .05* | -.01 | -.04 | -.15** |  |
| Subjective health | .44** | -.07** | -.12** | -.02 | -.24** | .21** |  |
| Depressive symptoms | -.35** | .08** | .11** | .04 | .09** | -.15** |  |
| Smoking status | -.33** | .06* | .07** | .02 |  | -.11** |  |
| Alcohol consumption | .29** | -.07** | -.07** | -.03 |  | .26** |  |
| Immediate memory | .25** | -.01 | -.05** | -.06* | -.34** | .24** |  |
| Verbal fluency | .25** | -.01 | -.04* | -.08** | -.28** | .23** |  |
| Education | .01 | .01 | .01 | -.25** | -.17** |  |  |
| ** *p*-value < or = .001, * *p*-value < or = .01 | | | | | | |  |
| *N* = 12,653, CFI = .959, RMSEA = .041 | | | | | | |  |

**Table J**

*Standardized Coefficients of the Indirect Effect of Early Adversity on Health, Lifestyle, and Cognition via Education in the ELSA Dataset*

| **Mediations** | **Standardized coefficients** | | **Standard Error** | ***z*** | ***p*-value** | **95% confidence interval** | |
| --- | --- | --- | --- | --- | --- | --- | --- |
|  |  |  | |  |  | **Lower limit** | **Upper limit** |
| physical assault - education - BMI | -.001 | .01 | | -.17 | .86 | -.02 | .01 |
| physical assault - education - self-rated health | .002 | .01 | | .17 | .86 | -.02 | .02 |
| physical assault - education - depression | -.001 | .01 | | -.17 | .86 | -.02 | .01 |
| physical assault - education - smoking | -.001 | .01 | | -.17 | .86 | -.01 | .01 |
| physical assault - education - alcohol | .002 | .01 | | .17 | 86 | -.02 | .03 |
| physical assault - education - immediate memory | .002 | .01 | | .17 | .86 | -.02 | .03 |
| physical assault - education – verbal fluency | .002 | .01 | | .17 | .86 | -.02 | .03 |
| sexual assault - education - BMI | -.002 | .003 | | -.58 | .56 | -.01 | .004 |
| sexual assault - education - self-rated health | .002 | .004 | | .58 | .57 | -.005 | .01 |
| sexual assault - education - depression | -.002 | .003 | | -.57 | .57 | -.01 | .004 |
| sexual assault - education - smoking | -.001 | .002 | | -.57 | .57 | -.005 | .003 |
| sexual assault - education - alcohol | .003 | .005 | | .58 | .56 | -.006 | .01 |
| sexual assault - education - immediate memory | .002 | .004 | | .59 | .56 | -.006 | .01 |
| sexual assault - education - verbal fluency | .002 | .004 | | .59 | .56 | -.01 | .01 |
| parental abuse - education - BMI | -.001 | .002 | | -.48 | .63 | -.01 | .004 |
| parental abuse - education - self-rated health | .002 | .004 | | .48 | .63 | -.005 | .01 |
| parental abuse - education - depression | -.001 | .002 | | -.47 | .64 | -.01 | .004 |
| parental abuse - education - smoking | -.001 | .002 | | -.47 | .64 | -.005 | .003 |
| parental abuse - education - alcohol | .002 | .004 | | .48 | .63 | -.01 | .01 |
| parental abuse - education - immediate memory | .002 | .004 | | .48 | .63 | -.01 | .01 |
| parental abuse - education – verbal fluency | .002 | .004 | | .48 | .63 | -.01 | .01 |
| deprivation - education - BMI | .036 | .005 | | 7.48 | <.001 | .03 | .05 |
| deprivation - education - self-rated health | -.053 | .01 | | -7.05 | <.001 | -.07 | -.04 |
| deprivation - education - depression | .036 | .01 | | 5.89 | <.001 | .02 | .05 |
| deprivation - education - smoking | .028 | .01 | | 4.67 | <.001 | .02 | .04 |
| deprivation - education - alcohol | -.063 | .01 | | -9.74 | <.001 | -.08 | -.05 |
| deprivation - education - immediate memory | -059 | .01 | | -10.23 | <.001 | -.07 | -.05 |
| deprivation - education – verbal fluency | -.058 | .01 | | -10.06 | <.001 | -.07 | -.05 |

**Table K**

*Cross Tabulation and Chi^2^ Independence Test between Physical Neglect and Sexual Abuse in the UK Biobank Dataset*

| **Physical neglect** | **Sexual abuse** | |  |
| --- | --- | --- | --- |
|  | No | Yes | Total |
| No | 119,378 | 9,914 | 129,292 |
| Yes | 21,540 | 3,642 | 25,182 |
| Total | 140,918 | 13,556 | 154,474 |
| Pearson chi^2^(1) = 1.2e+03, *p*-value < .001 | | | |

**Table L**

*Cross Tabulation and Chi^2^ Independence Test between Physical Neglect and Emotional Neglect in the UK Biobank Dataset*

| **Physical neglect** | **Emotional neglect** | |  |
| --- | --- | --- | --- |
|  | No | Yes | Total |
| No | 77,006 | 53,136 | 130,142 |
| Yes | 4,235 | 21,330 | 25,565 |
| Total | 81,241 | 74,466 | 155,707 |
| Pearson chi^2^(1) = 1.6e+04^,^ *p*-value < .001 | | | |

**Table M**

*Cross Tabulation and Chi^2^ Independence Test between Physical Neglect and Physical Abuse in the UK Biobank Dataset*

| **Physical neglect** | **Physical abuse** | |  |
| --- | --- | --- | --- |
|  | No | Yes | Total |
| No | 108,423 | 21,914 | 130,337 |
| Yes | 17,984 | 7,629 | 25,613 |
| Total | 126,407 | 29,543 | 155,950 |
| Pearson chi^2^(1) = 2.3e+03, *p*-value < .001 | | | |

**Table N**

*Cross Tabulation and Chi^2^ Independence Test between Physical Neglect and Emotional Abuse in the UK Biobank Dataset*

| **Physical neglect** | **Emotional abuse** | |  |
| --- | --- | --- | --- |
|  | No | Yes | Total |
| No | 113,457 | 16,836 | 130,293 |
| Yes | 18,084 | 7,484 | 25,568 |
| Total | 131,541 | 24,320 | 155,861 |
| Pearson chi^2^(1) = 4.3e+03, *p*-value < .001 | | | |

**Table O**

*Cross Tabulation and Chi^2^ Independence Test between Sexual Abuse and Emotional Neglect in the UK Biobank Dataset*

| **Sexual abuse** | **Emotional neglect** | |  |
| --- | --- | --- | --- |
|  | No | Yes | Total |
| No | 76,089 | 65,230 | 141,319 |
| Yes | 4,810 | 8,784 | 13,594 |
| Total | 80,899 | 74,014 | 154,913 |
| Pearson chi^2^(1) = 1.7e+03, *p*-value < .001 | | | |

**Table P**

*Cross Tabulation and Chi^2^ Independence Test between Sexual Abuse and Physical Abuse in the UK Biobank Dataset*

| **Sexual abuse** | **Physical abuse** | |  |
| --- | --- | --- | --- |
|  | No | Yes | Total |
| No | 117,432 | 24,147 | 141,579 |
| Yes | 8,538 | 5,064 | 13,602 |
| Total | 125,970 | 29,211 | 155,181 |
| Pearson chi^2^(1) = 3.3e+03, *p*-value < .001 | | | |

**Table Q**

*Cross Tabulation and Chi^2^ Independence Test between Sexual Abuse and Emotional Abuse in the UK Biobank Dataset*

| **Sexual abuse** | **Emotional abuse** | |  |
| --- | --- | --- | --- |
|  | No | Yes | Total |
| No | 122,196 | 19,324 | 141,520 |
| Yes | 8,942 | 4,641 | 13,583 |
| Total | 131,138 | 23,965 | 155,103 |
| Pearson chi^2^(1) = 4.0e+03, *p*-value < .001 | | | |

**Table R**

*Cross Tabulation and Chi^2^ Independence Test between Emotional Neglect and Physical Abuse in the UK Biobank Dataset*

| **Emotional neglect** | **Physical abuse** | |  |
| --- | --- | --- | --- |
|  | No | Yes | Total |
| No | 73,119 | 8,377 | 81,496 |
| Yes | 53,626 | 21,289 | 74,915 |
| Total | 126,745 | 29,666 | 156,411 |
| Pearson chi^2^(1) = 8.4e+03, *p*-value < .001 | | | |

**Table S**

*Cross Tabulation and Chi^2^ Independence Test between Emotional Neglect and Emotional Abuse in the UK Biobank Dataset*

| **Emotional neglect** | **Emotional abuse** | |  |
| --- | --- | --- | --- |
|  | No | Yes | Total |
| No | 76,897 | 4,612 | 81,509 |
| Yes | 54,981 | 19,845 | 74,826 |
| Total | 131,878 | 24,457 | 156,335 |
| Pearson chi^2^(1) = 1.3e+04, *p*-value < .001 | | | |

**Table T**

*Cross Tabulation and Chi^2^ Independence Test between Physical Abuse and Emotional Abuse in the UK Biobank Dataset*

| **Physical abuse** | **Emotional abuse** | |  |
| --- | --- | --- | --- |
|  | No | Yes | Total |
| No | 114,868 | 12,140 | 127,008 |
| Yes | 17,308 | 12,302 | 29,610 |
| Total | 132,176 | 24,442 | 156,618 |
| Pearson chi^2^(1) = 1.9e+04, *p*-value < .001 | | | |

**Table U**

*Standardized Coefficients of the Model for Male UK Biobank*

| **UKB Dataset** | | | | | | | |
| --- | --- | --- | --- | --- | --- | --- | --- |
| **Outcomes** | **Early adversity** | | | | | **Control variables** | |
|  | Physical abuse | Emotional abuse | Physical neglect | Sexual abuse | Emotional neglect | Age | Education |
| BMI | .020 | .075* | .005 | .021 | -.001 | -.097** | -.163** |
| Subjective health | .003 | -.054 | -.049 | -.027 | -.073* | .047 | .087** |
| Depressive symptoms | .046** | .125** | .013** | .057** | .074** | -.146** | -.036** |
| Smoking status | .008 | .067* | .030 | -.028 | -.023 | - | -.004 |
| Alcohol consumption | -.002 | -.016 | -.034 | -.030 | -.006 | - | .064* |
| Errors in visual memory | -.006 | .007 | .025** | .003 | -.003 | .139** | -.013* |
| Trail Making Test (Switching) | .003 | .003 | .114** | .005 | .0004 | .368** | -.198** |
| Reaction time | -.016 | .029 | .016 | .050 | .006 | .357** | -.059* |
| Fluid intelligence | -.004 | .009 | -.127** | -.007 | .007 | -.093** | .296** |
| ** *p*-value < or = .001, * *p*-value < or = .01  *N* = 229,061  RMSEA = .005  CFI = .995 | | | | | | | |

**Table V**

*Standardized Coefficients of the Model for Female UK Biobank*

| **UKB Dataset** | | | | | | | |
| --- | --- | --- | --- | --- | --- | --- | --- |
| **Outcomes** | **Early adversity** | | | | | **Control variables** | |
|  | Physical abuse | Emotional abuse | Physical neglect | Sexual abuse | Emotional neglect | Age | Education |
| BMI | .050 | -.002 | .036 | .002 | .023 | -.047 | -.113** |
| Subjective health | .009 | -.081** | .013 | -.036 | -.076** | .016 | .091** |
| Depressive symptoms | .038** | .127** | .007 | .061** | .091** | -.134** | -.052** |
| Smoking status | -.004 | .016 | -.027 | -.016 | .030 | - | -.008 |
| Alcohol consumption | -.041 | -.018 | -.054 | .005 | .0002 | - | .056* |
| Errors in visual memory | -.003 | -.001 | .018** | .008 | .004 | .118** | -.016** |
| Trail Making Test (Switching) | .006 | -.002 | .079** | .010 | .013* | .377** | -.128** |
| Reaction time | -.014 | .028 | .024 | .010 | -.023 | .367** | -.084** |
| Fluid intelligence | -.013* | .014** | -.099** | -.006 | -.001 | -.058** | .313** |
| ** *p*-value < or = .001, * *p*-value < or = .01  *N* = 273,299  RMSEA = .004  CFI = .997 | | | | | | | |

**Table W**

*Standardized Coefficients of the Mediation Model in the UK Biobank Dataset*

| **UKB Dataset** | | | | | | | |
| --- | --- | --- | --- | --- | --- | --- | --- |
| **Outcomes** | **Early adversity** | | | | | **Control variables** | |
|  | Physical abuse | Emotional abuse | Physical neglect | Sexual abuse | Emotional neglect | Age | Education |
| BMI | .04 | .02 | .02 | -.003 | .02 | -.06** | -.13** |
| Subjective health | .01 | -.07** | -.01 | -.03 | -.08** | .03 | .09** |
| Depressive symptoms | .03** | .13** | .01** | .07** | .08** | -.15** | -.05** |
| Smoking status | .002 | .04 | -.001 | -.02 | .004 |  | -.01 |
| Alcohol consumption | -.02 | -.03 | -.05* | -.03 | .01 |  | .06** |
| Errors in visual memory | -.004 | .002 | .02** | .01 | .001 | .13** | -.01** |
| Trail Making Test (Switching) | .0004 | .004 | .10** | .01** | .004 | .37** | -.16** |
| Reaction time | -.02 | .03 | .03 | .04 | -.01 | .35** | -.08** |
| Fluid intelligence | -.004 | .008 | -.11** | -.01** | .01 | -.07** | .31** |
| Education | -.03** | .02** | -.11** | .01** | -.02** | -.17** |  |
| ** *p*-value < or = .001, * *p*-value < or = .01 | | | | | | | |
| *N* = 502,360, CFI = .997, RSMEA = .004 | | | | | | | |

**Table X**

*Standardized Coefficients of the Indirect Effect of Early Adversity on Health, Lifestyle, and Cognition via Education in the UK Biobank Dataset*

| **Mediations** | **Standardized coefficients** | **Standard Error** | ***z*** | ***p*-value** | **95% confidence interval** | |
| --- | --- | --- | --- | --- | --- | --- |
|  |  |  |  |  | **Lower limit** | **Upper limit** |
| physical abuse - education - BMI | .004 | .001 | 6.76 | <.001 | .003 | .01 |
| physical abuse - education - self-rated health | -.003 | .0005 | -5.30 | <.001 | -.004 | -.002 |
| physical abuse - education - depression | .001 | .0002 | 9.06 | <.001 | .001 | .002 |
| physical abuse - education - smoking | .0002 | .0005 | 0.39 | .694 | -.001 | .001 |
| physical abuse - education - alcohol | -.002 | .0005 | -3.89 | <.001 | -.003 | -.001 |
| physical abuse - education - visual episodic memory | .0004 | .0001 | 4.19 | <.001 | .0002 | .0006 |
| physical abuse - education - TMTB | .005 | .0005 | 10.29 | <.001 | .004 | .006 |
| physical abuse - education - fluid intelligence | -.01 | .001 | -10.44 | <.001 | -.01 | -.01 |
| physical abuse - education - processing speed | .002 | .0005 | 4.76 | <.001 | .001 | .003 |
| emotional abuse - education - BMI | -.003 | .0005 | -5.64 | <.001 | -.004 | -002 |
| emotional abuse - education - self-rated health | .002 | .0004 | 4.70 | <.001 | .001 | .003 |
| emotional abuse - education - depression | -.001 | .0002 | -6.79 | <.001 | -.001 | -.001 |
| emotional abuse - education - smoking | -.0001 | .0003 | -.39 | .694 | -.001 | .001 |
| emotional abuse - education - alcohol | .001 | .0004 | 3.63 | <.001 | .001 | .002 |
| emotional abuse - education - visual episodic memory | -.0003 | .0001 | -3.88 | <.001 | -.0005 | -.0002 |
| emotional abuse - education – TMTB | -.003 | .0005 | -7.27 | <.001 | -.004 | -.003 |
| emotional abuse - education - fluid intelligence | .01 | .001 | 7.32 | <.001 | .005 | .01 |
| emotional abuse - education – processing speed | -.002 | .0004 | -4.32 | <.001 | -.002 | -.001 |
| physical neglect - education - BMI | .01 | .002 | 8.61 | <.001 | .01 | .02 |
| physical neglect - education - self-rated health | -.01 | .002 | -6.07 | <.001 | -.01 | -.01 |
| physical neglect - education - depression | .01 | .0003 | 16.30 | <.001 | .005 | .006 |
| physical neglect - education - smoking | .001 | .002 | 0.39 | .694 | -.003 | .004 |
| physical assault - education - alcohol | -.01 | .002 | -4.16 | <.001 | -.01 | -.004 |
| physical neglect - education - visual episodic memory | .002 | .0003 | 4.54 | <.001 | .001 | .002 |
| physical neglect - education – TMTB | .02 | .001 | 31.65 | <.001 | .02 | .02 |
| physical neglect - education – fluid intelligence | -.03 | .001 | -36.54 | <.001 | -.03 | -.03 |
| physical neglect - education – processing speed | .01 | .002 | 5.30 | <.001 | .01 | .01 |
| sexual abuse - education - BMI | -.002 | .0004 | -4.14 | <.001 | -.002 | -.001 |
| sexual abuse - education - self-rated health | .001 | .0003 | 3.73 | <.001 | .001 | .002 |
| sexual abuse - education - depression | -.001 | .0001 | -4.54 | <.001 | -.001 | -.0003 |
| sexual abuse - education - smoking | -.0001 | .0002 | -.039 | .695 | -.0005 | .0003 |
| sexual abuse - education - alcohol | .001 | .0003 | 3.12 | .002 | .0003 | .001 |
| sexual abuse - education - visual episodic memory | -.0002 | .0001 | -3.27 | .001 | -.0003 | -.0001 |
| sexual assault - education - TMTB | -.002 | .0004 | -4.67 | <.001 | -.003 | -.001 |
| sexual assault - education - fluid intelligence | .004 | .001 | 4.68 | <.001 | .002 | .01 |
| sexual assault - education - processing speed | -.001 | .0003 | -3.52 | <.001 | -.002 | -.0004 |
| emotional neglect - education - BMI | .003 | .001 | 6.17 | <.001 | .002 | .004 |
| emotional neglect - education - self-rated health | -.002 | .0004 | -5.01 | <.001 | -.003 | -.001 |
| emotional neglect - education - depression | .001 | .0002 | 7.76 | <.001 | .001 | .002 |
| emotional neglect - education - smoking | .0001 | .0004 | 0.39 | .694 | -.001 | .001 |
| emotional neglect - education - alcohol | -.002 | .0004 | -3.76 | <.001 | -.002 | -.001 |
| emotional neglect - education - visual episodic memory | .0004 | .0001 | 4.04 | <.001 | .0002 | .001 |
| emotional neglect - education –TMTB | .004 | .0005 | 8.50 | <.001 | .003 | .01 |
| emotional neglect - education - fluid intelligence | -.01 | .001 | -8.57 | <.001 | -.01 | -.01 |
| emotional neglect - education – processing speed | .002 | .0004 | 4.54 | <.001 | .001 | .003 |
